# Notch1 Signaling Regulates the Th17/Treg Immune Imbalance in Patients with Psoriasis Vulgaris

**DOI:** 10.1155/2018/3069521

**Published:** 2018-03-04

**Authors:** Lei Ma, HaiBo Xue, Tianqin Gao, MeiLan Gao, YuJie Zhang

**Affiliations:** ^1^Department of Dermatology, Binzhou Medical University Hospital, 661 Second Huanghe Road, Binzhou 256603, China; ^2^Department of Endocrinology and Metabolism, Binzhou Medical University Hospital, 661 Second Huanghe Road, Binzhou 256603, China; ^3^Department of Operating Room, Binzhou Medical University Hospital, 661 Second Huanghe Road, Binzhou 256603, China; ^4^Department of Laboratory, Binzhou Medical University Hospital, 661 Second Huanghe Road, Binzhou 256603, China

## Abstract

**Purpose:**

To evaluate the regulating effect of Notch1 signaling on Th17/Treg immune imbalance in psoriasis vulgaris (PV).

**Materials and Methods:**

Notch1, Hes-1, ROR*γ*t, Foxp3, IL-17, and IL-10 mRNA expression, as well as Th17 and Treg cell percentages in peripheral CD4^+^ T cells, were detected by real-time quantitative RT-PCR and flow cytometry, and serum concentrations of IL-17 and IL-10 were detected by ELISA in 36 PV patients and 32 healthy controls. Additionally, CD4^+^ T cells from 12 PV patients were treated with *γ*-secretase inhibitor DAPT, and the above indexes were measured.

**Results:**

PV patients presented distinct Th17/Treg immune imbalance and highly expressed Notch1 and Hes-1 mRNA levels, which were positively correlated with psoriasis area and severity index (PASI) and the ratios of Th17/Treg and ROR*γ*t/Foxp3. DAPT treatment resulted in the obvious downregulation of Th17 cell percentage in cocultured CD4^+^ T cells, ROR*γ*t and IL-17 mRNA levels, and IL-17 concentration in cell-free supernatant from cocultured CD4^+^ T cells of PV patients in a dose-dependent manner, while there was no significant influence on Treg cell percentage, Foxp3, and IL-10 expression, therefore leading to the recovery of Th17/Treg immune imbalance.

**Conclusion:**

Notch1 signaling may contribute to the pathogenesis of PV by regulating Th17/Treg immune imbalance.

## 1. Introduction

Psoriasis is a CD4^+^ T cell-medicated autoimmune and inflammatory cutaneous disorder, which affects 2% to 3% of the world population [1]. Psoriasis can be divided into four types, among which psoriasis vulgaris (PV) is the most common type, accounting for 90% of all cases. IL-17-expressing T cells (termed Th17) and CD4^+^CD25^+^Foxp3^+^ regulatory T cells (termed Treg) are newly defined CD4^+^ T cell subsets, which have opposite effects on autoimmunity and inflammation. Th17 cells play a crucial role in the pathogenesis and development of autoimmune and inflammatory reactions by producing high levels of IL-17. ROR*γ*t is the specific transcription factor of Th17 cells, which is essential for Th17 development and function [2, 3]. Treg cells, characterized by high expression of the transcription factor Foxp3, are considered to be important for maintaining self-tolerance and preventing autoimmune and inflammatory diseases by directly contacting effective immune cells; releasing suppressive cytokines, such as IL-10 and transforming growth factor- (TGF-) *β*; and exhibiting their immunosuppressive effects on T cells [4–6]. Th17/Treg immune imbalance has been proved to widely exist in autoimmune and inflammatory diseases [7–9]. Recently, the possible roles of Th17 and Treg cells in the pathogenesis of PV have been reported; however, the results were not completely consistent [10–13].

Notch signaling is an evolutionarily conserved cell-to-cell signaling cascade involved in cell differentiation, proliferation, and fate decision processes in multiple organisms and tissues, including early T cell development in the thymus and modulation of peripheral T cell differentiation [14–16]. Notch signaling is initiated when Notch receptors are engaged with a Notch ligand. In mammals, four Notch receptors (Notch1–4) and five Notch ligands (Delta-like 1, 3, and 4; Jagged1 and 2) have been identified [17]. *γ*-Secretase inhibitors are able to effectively inhibit Notch receptor signaling and result in the inhibition of the release of an active intracellular domain (Notch intracellular domain, NICD), which can translocate to the nucleus, associate with transcription factors, and modulate target genes' expression and cell growth and development. It has been reported that Notch signaling may play a decisive role in the differentiation of T cells into the initial stage of Th17 and/or Treg cells [18], and some studies have also indicated the role of Notch signaling in the regulation of Th17 cell differentiation and function as well as its recovery effect on Th17/Treg immune imbalance in some autoimmune and inflammatory diseases [19–23].

In this study, we detected the expression and relationship of Notch1 and its target gene Hes-1 with Th17/Treg immune imbalance and the disease severity in patients with PV and explored the recovery effect of Notch1 signaling inhibition on the Th17/Treg immune imbalance of PV.

## 2. Materials and Methods

### 2.1. Patients and Controls

36 newly diagnosed PV patients (20 males and 16 females, aged 18–46 years) were enrolled in the study, and the disease severity was evaluated by psoriasis area and severity index (PASI). Meanwhile, 32 age- and sex-matched healthy volunteers were selected as the healthy controls. This study was approved by the Ethics Committee of Binzhou Medical University Hospital. Written informed consents were obtained from all participants.

### 2.2. Blood Sample Collection and Preparation

10 ml peripheral venous blood was collected, of which a 3 ml sample was used for the detection of serum concentrations of cytokines, and the other 7 ml sample was used for CD4^+^ T cell isolation. According to the manufacturer's instructions, peripheral blood mononuclear cells (PBMCs) were separated by Ficoll-Hypaque density gradient centrifugation (Amersham Biosciences, Piscataway, NJ, USA), and untouched CD4^+^ T cells were isolated from PBMCs by immunomagnetic beads (Miltenyi Biotec, Auburn, CA, USA). The purity of the CD4^+^ T cells was 94.1 ± 1.6% as evaluated by flow cytometry, and the cell vitality was 95.6 ± 1.5% as assessed by trypan blue staining. CD4^+^ T cells were harvested for flow cytometric analysis, RNA extraction, and *γ*-secretase inhibitor DAPT treatment.

### 2.3. Flow Cytometric Analysis of Th17 and Treg Cell Percentages

For the analysis of Th17 cell percentage (CD4^+^IL17^+^ T cells/CD4^+^ T cells%), CD4^+^ T cells were firstly stimulated with 25 ng/ml phorbol myristate acetate (PMA) and 1 *μ*g/ml ionomycin in the presence of 2 mmol/ml monensin at 37°C under a 5% CO_2_ environment for 5 hours. Next, cells were stained with FITC-labeled CD4 antibody at 4°C in the dark for 30 minutes. After surface staining, cells were fixed, permeabilized, and then stained with PE-labeled IL-17 antibody.

For the analysis of Treg cell percentage (CD4^+^CD25^+^Foxp3^+^ T cells/CD4^+^ T cells%), CD4^+^ T cells were firstly stained to the surface with FITC-labeled CD4 and APC-labeled CD25 antibodies at 4°C in the dark for 30 minutes. For further Foxp3 intracellular staining, cells were fixed and permeabilized by a commercial cell fixation/permeabilization kit and then stained with PE-labeled Foxp3 antibody.

All flow cytometry antibodies were from the eBioscience company (San Diego, CA, USA), and other reagents were from the Sigma-Aldrich company (St. Louis, MO, USA) and the BD Biosciences company (San Jose, CA, USA). Flow cytometric analysis was performed on a FACSCanto flow cytometer (BD Biosciences). Isotype controls were used to correct nonspecific binding in all procedures.

### 2.4. Real-Time Quantitative RT-PCR Analysis of Notch1, Hes-1, ROR*γ*t, IL-17, Foxp3, and IL-10 mRNA Expression Levels

Total RNA was isolated from CD4^+^ T cells with TRIzol reagent (Invitrogen, Carlsbad, CA, USA). The quality of RNA samples was assessed by the 260/280 absorbance ratio, which ranged from 1.9 to 2.0. And clear 28S and 18S bands after 1.5% agarose gel electrophoresis were observed for each RNA sample. Complementary DNA was synthesized by the PrimeScript™ RT reagent kit (Toyobo, Osaka, Japan). The expression levels of Notch1, Hes-1, ROR*γ*t, IL-17, Foxp3, and IL-10 mRNA were quantified by SYBR Premix Ex Taq™ II (TaKaRa) with the given primers (Table 1) on a Rotor-Gene 3000 (Corbett Research, Sydney, Australia) and analyzed by the relative standard curve method.

### 2.5. Enzyme-Linked Immunosorbent Assay for IL-17 and IL-10

Serum was isolated within 30 minutes of blood collection and stored at −80°C for further measurement of cytokine levels. The serum concentrations of IL-17 and IL-10 were detected by enzyme-linked immunosorbent assay (ELISA) kits (R&D Systems, Minneapolis, MN, USA).

### 2.6. CD4^+^ T Cell Culture and *γ*-Secretase Inhibitor DAPT Treatment

To evaluate the effect of DAPT on Th17 cell differentiation, CD4^+^ T cells from 12 PV patients were treated with DAPT (Sigma-Aldrich) at desired concentrations of 2.5 *μ*mol/l, 5 *μ*mol/l, 10 *μ*mol/l, and 20 *μ*mol/l. DAPT was dissolved in dimethyl sulfoxide (DMSO, Sigma-Aldrich), and the DMSO final concentration never exceeded 0.1%. CD4^+^ T cells treated with DMSO only were used as controls. CD4^+^ T cells were collected after incubation in the presence of DAPT or DMSO at 37°C and 5% CO_2_ environment for 72 hours.

### 2.7. Th17 Cell Polarization

After DAPT pretreatment, CD4^+^ T cells were treated under Th17 cell-polarizing environment, including 5 *μ*g/ml CD3 monoclonal antibody (mAb), 2 *μ*g/ml CD28 mAb, 10 ng/ml recombinant IL- (rIL-) 1*β*, 50 ng/ml rIL-6, 20 ng/ml rIL-23, 10 *μ*g/ml anti-interferon- (IFN-) *γ* antibody, and 10 *μ*g/ml anti-IL-4 antibody for 96 hours. Antibodies and recombinant cytokines were all from the eBioscience company. Then, cells were gathered and followed by PMA and ionomycin stimulation for flow cytometric detection. RNA samples and cell-free supernatant from polarized CD4^+^ T cells were harvested for further real-time quantitative RT-PCR and ELISA analysis, respectively.

### 2.8. CD4^+^ T Cell Viability Detection

The effect of DAPT treatment on CD4^+^ T cell proliferation was determined by the Cell Counting Kit-8 (CCK-8) from Dojindo Laboratories (Kumamoto, Japan) and measured by optical density at 450 nm.

## 3. Statistical Analysis

According to the results of a normal distribution test, data were expressed as mean ± standard deviation, and independent-sample *t*-tests, Pearson's correlation coefficients, and one-way analysis of variance (ANOVA) were used for statistical analysis. All tests were performed by SPSS 17.0 and GraphPad Prism 5 systems. A *P*-value of <0.05 was considered statistically significant.

## 4. Results

### 4.1. Th17 and Treg Cell Percentages in Peripheral CD4^+^ T Cells

**Figure 1:**
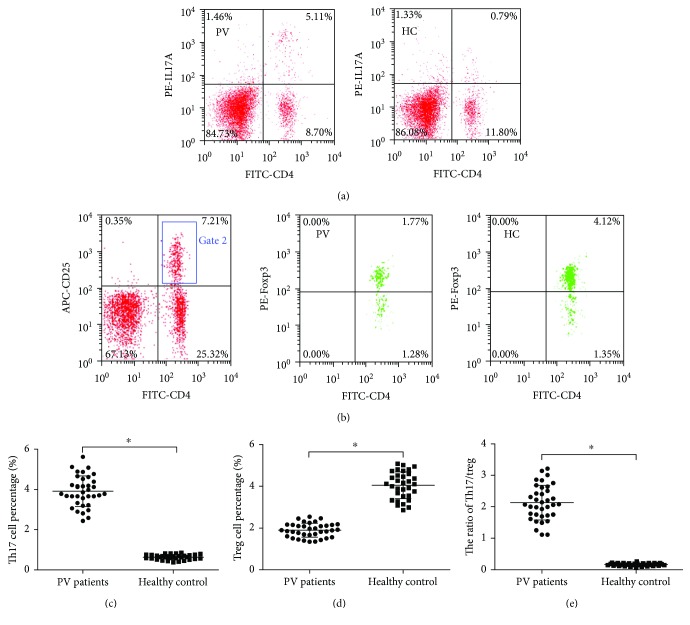
Th17 cell percentage, Treg cell percentage, and the ratio of Th17/Treg in CD4^+^ T cells of PV patients and healthy control. (a) Representative pictures for Th17 cell percentage (CD4^+^IL17^+^/CD4^+^ T cells%) of PV patients and healthy controls. (b) Representative pictures for Treg cell percentage (CD4^+^CD25^+^Foxp3^+^/CD4^+^ T cells%) of PV patients and healthy controls. (c) Th17 cell percentage of PV patients was much higher than healthy controls (^∗^*P* < 0.001). (d) PV patients had much lower Treg cell percentage than healthy controls (^∗^*P* < 0.001). (e) The ratio of Th17/Treg of PV patients increased significantly compared to healthy controls (^∗^*P* < 0.001).

Representative pictures for Th17 cell percentage (CD4^+^IL17^+^/CD4^+^ T cells%) and Treg cell percentage (CD4^+^CD25^+^Foxp3^+^/CD4^+^ T cells%) are presented in Figures 1(a) and 1(b). Th17 cell percentage in peripheral CD4^+^ T cells was significantly higher in PV patients than healthy controls (3.91 ± 0.76% versus 0.63 ± 0.13%, *t* = 25.549, *P* < 0.001, Figure 1(c)). In contrast, Treg cell percentage was dramatically decreased in PV patients compared with healthy controls (1.90 ± 0.33% versus 4.05 ± 0.64%, *t* = −17.041, *P* < 0.001, Figure 1(d)). As a result, the ratio of Th17/Treg was obviously increased in PV patients (2.13 ± 0.55 versus 0.16 ± 0.04, *t* = 21.314, *P* < 0.001, Figure 1(e)).

### 4.2. Notch1, Hes-1, ROR*γ*t, IL-17, Foxp3, and IL-10 mRNA Expression Levels in Peripheral CD4^+^ T Cells

**Figure 2:**
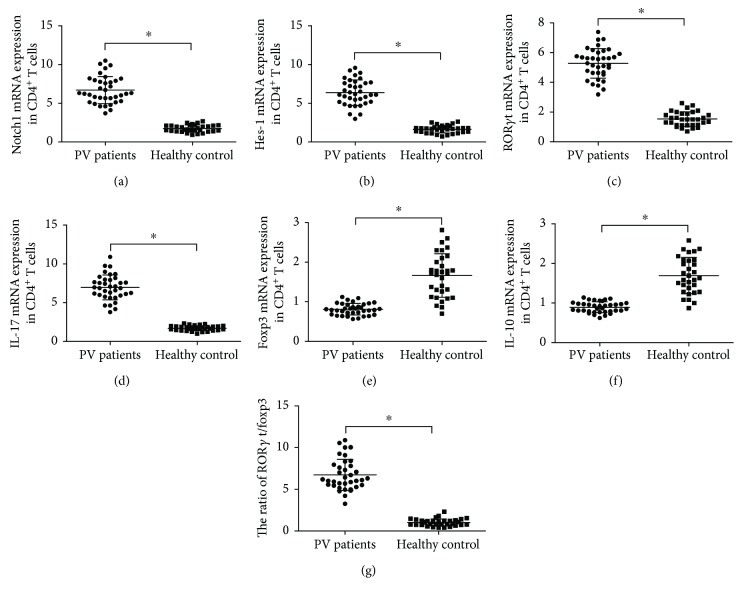
The mRNA expressions of Notch1 (a), Hes-1 (b), ROR*γ*t (c), and IL-17 (d) and the ratio of ROR*γ*t/Foxp3 (g) in PV patients increased significantly compared to those in healthy controls (^∗^*P* < 0.001). Meanwhile, PV patients presented lower Foxp3 (e) and IL-10 (f) mRNA expressions than healthy controls (^∗^*P* < 0.001).

Compared with healthy controls, mRNA expression levels of Notch1, Hes-1, ROR*γ*t, and IL-17 in peripheral CD4^+^ T cells of PV patients were obviously increased (Notch1, 6.70 ± 1.75 versus 1.73 ± 0.46, *t* = 16.395; Hes-1, 6.37 ± 1.64 versus 1.63 ± 0.49, *t* = 16.517; ROR*γ*t, 5.28 ± 1.00 versus 1.53 ± 0.49, *t* = 20.092; IL-17, 6.96 ± 1.61 versus 1.70 ± 0.33, *t* = 19.212; all *P* < 0.001, Figures 2(a)–2(d)), while mRNA expression levels of Foxp3 and IL-10 in PV patients were sharply decreased (Foxp3, 0.81 ± 0.15 versus 1.66 ± 0.55, *t* = −8.512; IL-10, 0.89 ± 0.14 versus 1.69 ± 0.46, *t* = −9.732; both *P* < 0.001, Figures 2(e) and 2(f)). In addition, the ratio of ROR*γ*t/Foxp3 was obviously higher in PV patients than in healthy controls (6.72 ± 1.87 versus 1.04 ± 0.58, *t* = 17.360, *P* < 0.001; Figure 2(g)).

### 4.3. Serum Concentrations of IL-17 and IL-10

**Figure 3:**
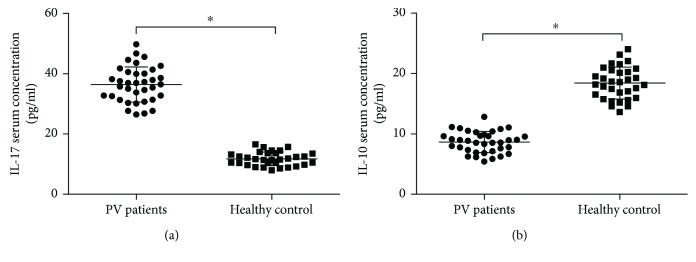
Serum IL-17 concentrations (a) in PV patients were much higher than those in healthy controls (^∗^*P* < 0.001). However, IL-10 concentrations (b) in PV patients were decreased obviously compared to those in healthy controls (^∗^*P* < 0.001).

IL-17 serum concentration was significantly elevated in PV patients (36.41 ± 5.85 pg/ml) in comparison with that in healthy controls (11.76 ± 2.26 pg/ml, *t* = 23.385, *P* < 0.001, Figure 3(a)), while IL-10 serum concentration was reduced (8.64 ± 1.76 pg/ml versus 18.43 ± 2.65 pg/ml, *t* = −17.736, *P* < 0.001, Figure 3(b)).

### 4.4. Correlation Analysis in PV Patients

**Figure 4:**
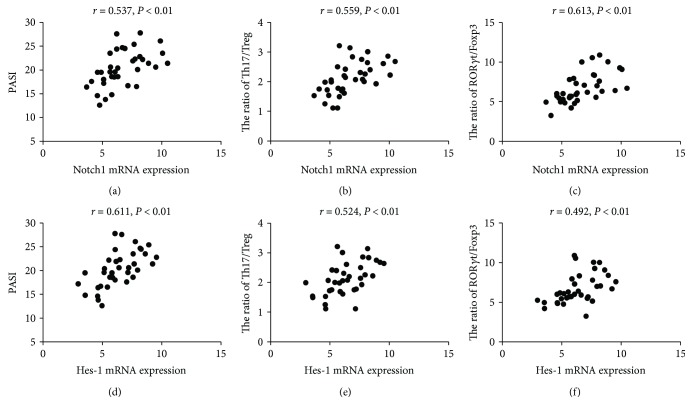
The correlation analysis of Notch1 and Hes-1 mRNA expression levels with psoriasis area and severity index (PASI) and the ratios of Th17/Treg and ROR*γ*t/Foxp3. Notch1 mRNA expressions positively correlated with PASI (a), the ratio of Th17/Treg (b), and the ratio of ROR*γ*t/Foxp3 (c). The increasing expressions of Hes-1 mRNA also had positive correlations with PASI (d), the ratio of Th17/Treg (e), and the ratio of ROR*γ*t/Foxp3 (f).

Both Notch1 and Hes-1 mRNA expression levels were positively correlated with PASI and the ratios of Th17/Treg and ROR*γ*t/Foxp3 (Notch1 mRNA, *r* = 0.537, 0.559, and 0.613, all *P* < 0.01, Figures 4(a)–4(c); Hes-1 mRNA, *r* = 0.611, 0.524, and 0.492, all *P* < 0.01, Figures 4(d)–4(f)).

### 4.5. DAPT Reduced the Abnormally Increased Th17 Cell Percentage and Th17/Treg Ratio in PV Patients

**Figure 5:**
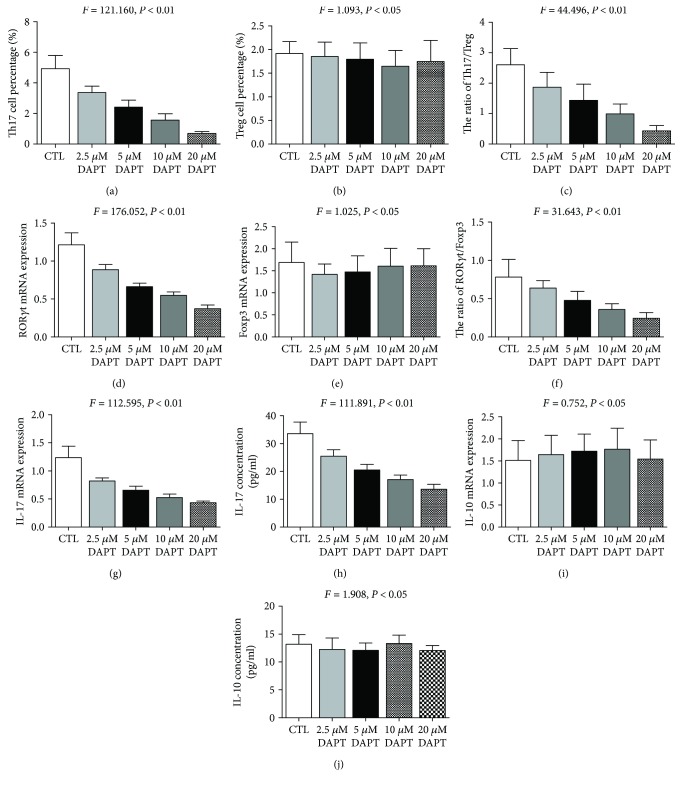
The percentages of Th17 cells (a), ROR*γ*t mRNA (d), and IL-17 mRNA (g) expression in cultured CD4^+^ T cells, as well as IL-17 concentrations (h) in cell-free supernatant decreased obviously in a dose-dependent manner after DAPT treatment (all *P* < 0.01), and the same characteristics could be seen in the ratio of Th17/Treg (c) and the ratio of ROR*γ*t/Foxp3 (f). However, there was no significant difference in Treg cell percentage (b), Foxp3 mRNA expression (e), IL-10 mRNA expression (i), and IL-10 concentrations (j) after DAPT treatment (all *P* > 0.05).

After DAPT treatment, the percentage of Th17 cells in cultured CD4^+^ T cells of PV patients was obviously decreased in a dose-dependent manner (*F* = 121.160, *P* < 0.01, Figure 5(a)), while there was no significant difference in the percentage of Treg cells (*F* = 1.093, *P* > 0.05, Figure 5(b)), and then the increased ratio of Th17/Treg was gradually restored with the increased concentration of DAPT (*F* = 44.496, *P* < 0.01, Figure 5(c)).

### 4.6. DAPT Downregulated the Abnormally Elevated ROR*γ*t mRNA Expression and ROR*γ*t/Foxp3 Ratio in PV Patients

Similar to the result of Th17 and Treg cell percentages, DAPT treatment resulted in reduced ROR*γ*t mRNA expression in cultured CD4^+^ T cells of PV patients (*F* = 176.052, *P* < 0.01, Figure 5(d)), while there was no significant change in Foxp3 mRNA expression (*F* = 1.025, *P* > 0.05, Figure 5(e)). Hence, the ratio of ROR*γ*t/Foxp3 showed gradual decrease in a dose-related way (*F* = 31.643, *P* < 0.01, Figure 5(f)).

### 4.7. DAPT Decreased IL-17 mRNA Expression and Secretion in PV Patients

The IL-17 mRNA expression in cultured CD4^+^ T cells and IL-17 concentrations in cell-free supernatant from cultured CD4^+^ T cells of PV patients were gradually decreased with the increased dose of DAPT (*F* = 112.595 and 111.891, resp., both *P* < 0.01, Figures 5(g) and 5(h)), while there was no obvious change in IL-10 mRNA expression and cell-free supernatant concentration (*F* = 0.752 and 1.908, resp., both *P* > 0.05, Figures 5(i) and 5(j)).

### 4.8. DAPT Had No Effect on CD4^+^ T Cell Proliferation

**Figure 6:**
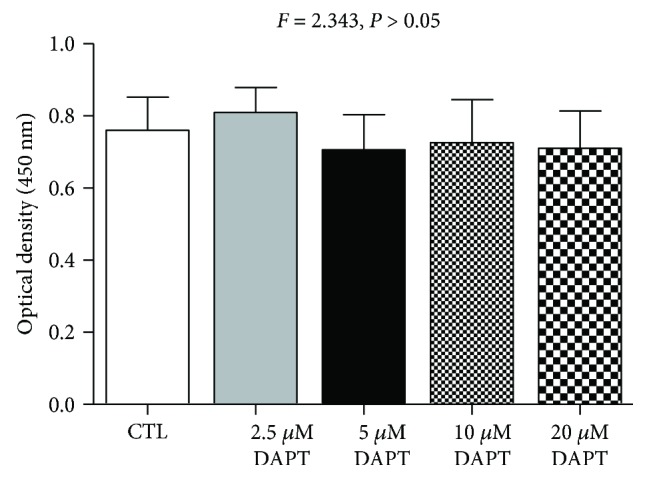
DAPT treatment did not affect CD4^+^ T cell proliferation of PV patients by CCK-8 assay.

CD4^+^ T cell growth of PV patients was determined by the CCK-8 assay after DAPT or DMSO treatment for 72 hours, and ANOVA revealed that there was no significant difference in the optical density values at 450 nm among DAPT-treated groups and control groups (*F* = 2.343, *P* > 0.05, Figure 6).

## 5. Discussion

Th17/Treg immune imbalance has been reported to contribute to the pathogenesis of many autoimmune and inflammatory diseases. In this study, our results provided direct evidence that PV patients presented a significant increase of circulating Th17 cell percentage, Th17-specific transcription factor ROR*γ*t mRNA expression, and its effective cytokine IL-17 mRNA expression and serum levels, while a dramatically decreased proportion of Treg cells and expression levels of Treg-specific transcription factor Foxp3 and its effective cytokine IL-10, which results in an imbalanced ratio of Th17/Treg and ROR*γ*t/Foxp3, then confirms that Th17/Treg immune imbalance exists in PV patients.

Notch signaling has been demonstrated to play a pivotal role in a broad spectrum of cellular activities, such as proliferation, differentiation, and regulation of cell function in many cell lineages [14–16, 24]. Hyperproliferative epidermis and mixed cutaneous lymphocytic infiltration are the characters of PV, and the abnormal immune microenvironment caused by Th17 cytokines IL-17 and IL-22 in psoriatic lesion modulates the distinct inflammatory and keratinocyte-response pathways [25]. Upregulated expression of Notch1 has been demonstrated in psoriatic lesions, which can mediate the abnormal differentiation of epidermal keratinocytes and implies the possible role of Notch1 in the pathogenesis of psoriasis [26, 27]. In this study, the elevated expression of Notch1 and its target gene Hes-1 was demonstrated in peripheral CD4^+^ T cells of PV patients and positively associated with psoriasis area and severity index, which indicates that Notch1 signaling participates in the development and progress of PV. Notch signaling has been considered as one of the main factors in regulating lymphocyte activation and differentiation, and the Th cell polarization and cytokine production may depend on Notch receptor-ligand interactions [28, 29]. Besides the increased expression of Notch1 and its target gene Hes-1 in psoriatic peripheral CD4^+^ T cells, we also found that both Notch1 and Hes-1 expression were positively correlated with the ratios of Th17/Treg and ROR*γ*t/Foxp3, which signifies a direct link between Notch1 and Th17/Treg immune imbalance in PV.

The molecular mechanism of Notch signaling is unique: ligands bind to the extracellular domain of Notch and trigger sequential proteolytic cleavages. Finally, *γ*-secretase releases NICD from the cell membrane, and NICD translocates to the nucleus. In the nucleus, NICD interacts with the DNA-binding protein known as CSL (CBF-1, suppressor of hairless, Lag-1) and modifies the gene expression. Thus, Notch activation is dependent on *γ*-secretase [30]. DAPT is a *γ*-secretase inhibitor and is commonly used to block Notch signaling [31]. Presenilin, a molecular target of DAPT, is an important component of the *γ*-secretase and critical for *γ*-secretase activity [32]. DAPT can effectively block presenilin and finally result in the inhibition of active NICD release. Two CSL binding sites have been confirmed within the human IL-17 promoter, upstream of the transcription start site. Notch1 can directly bind to these CSL binding sites in the human IL-17 promoter, and the binding can be inhibited by pretreatment with *γ*-secretase inhibitors [19]. Thus, Notch1 signaling can regulate IL-17 promoter activity and Th17 cell differentiation [19]. The blockade of Notch1 signaling by *γ*-secretase inhibitor resulted in the marked downregulation of Th17 cells, effective cytokine IL-17 secretion, and Th17-mediated disease progression in experimental autoimmune encephalomyelitis (EAE) [19]. In addition, the latest research demonstrated critical crosstalk between the IL-17 and Notch1 signaling [33]: IL-17R interacts with Notch1 via the extracellular domain, which facilitates the cleavage of NICD1, formation of the Act1-NICD1 complex, and subsequent translocation into the nucleus. Act1 is a key adaptor molecule in IL-17 signaling and plays a very important role in IL-17 signal transduction [34]. As a result, Act1-NICD1 is recruited to the promoters of several Th17-induced Notch1 target genes [33]. The ablation of Notch1 and disruption of the IL-17RA-Notch1 interaction can inhibit IL-17-induced Notch1 activation and attenuate Th17-mediated EAE [33]. In the present study, we found that the Th17 cell percentage, IL-17 mRNA expression, and secretion in cocultured CD4^+^ T cells of PV patients were all gradually decreased with the increased dose of DAPT treatment. So, we concluded that the interruption of the crosstalk between the IL-17 and Notch1 signaling may be a primary mechanism of the blockade of Notch1 signaling regulating Th17 cell differentiation and function by the specific inhibitor DAPT.

ROR*γ*t is the specific transcription factor of Th17 cells, which is required for the effective induction, development, and function of Th17 cells [2, 3]. Evidences from an in vitro study have also shown that both mouse and human ROR*γ*t expression can be significantly reduced by *γ*-secretase inhibitor treatment and/or Notch1-specific siRNA, which indicates that Notch1 can regulate the expression of ROR*γ*t [19]. In addition, the ROR*γ*t promoter is the direct transcriptional Notch target [19, 35]. Our data showed a coordinated downregulation of ROR*γ*t with decreased Th17 cell percentage and IL-17 production in a dose-dependent manner in CD4^+^ T cells of PV patients in the presence of DAPT, which provides further evidence that ROR*γ*t can regulate Th17 cells directly, and the inhibition of the Notch1 signaling can disrupt Th17 cell differentiation and function through the downregulation of ROR*γ*t expression in PV.

In agreement with other researches on the effect of Notch inhibition by DAPT on the differentiation and function of CD4^+^ and CD8^+^ T cells [21, 23, 36], we did not observe a significant delay in psoriatic CD4^+^ T cell proliferation in the presence of DAPT. In addition, the study of Keerthivasan et al. confirmed that the decrease in IL-17 secretion by the other two *γ*-secretase inhibitor compounds E and IL-CHO was not due to an effect on cell proliferation [19]. The report from Sauma et al. also showed that the number of IL-17-secreting CD8^+^ T cells (Tc17 cells) after 3 days of treatment by DAPT was very similar to the number of cells treated by control vehicle DMSO, namely, the differences in IL-17 expression and production were not because of a variation in the number of cells [36]. Similar research in chronic hepatitis C patients showed that DAPT treatment did not notably influence the proliferation of cultured PBMCs, and there were no significant differences in the absolute number of cells between DAPT and DMSO treatments [23]. So, combining previous reports and our results, we concluded that the inhibition of Notch signaling by DAPT can regulate Th17 cell differentiation and function without affecting its activation and proliferation status.

Treg cells can restrain excessive effector T cell responses and contribute to immune tolerance with high expression of the transcriptional factor Foxp3, which is required for the development of Treg cells and appears to control a genetic programme specifying this cell fate [12]. Treg cells have been reported to constitutively express genes encoding Notch receptors and ligands in human thymus, and peripheral Treg cells can also preferentially express Notch ligands [37, 38], while, with regard to the role of Notch signaling in Treg cells, contradictory results are present, including positive, negative, or no significant effect on Treg cell homeostasis and function. A previous study by Asano et al. reported that the blockade of Notch1 signaling with an anti-JAG1 or a blocking anti-Notch1 antibody inhibited Treg suppressor function in vitro [38]. Recently, Marcel and Sarin reported that Notch1 can control the survival and inhibitor activity of murine Treg cells through the regulation of autophagy [39]. Nonnuclear Notch1 activity is required for the activation of autophagy in activated Tregs in response to cytokine withdrawal, and the induction of LC3 puncta formation, a marker of autophagy, can be significantly interrupted by *γ*-secretase inhibitor treatment, ablation of Delta-like 1, and targeted deletion of Notch1. Moreover, NICD can form complexes with Beclin and Atg14, specific components of the autophagy pathway, which indicated a more direct role for Notch1 in the autophagic cascade in Tregs. The Notch-autophagy axis can control the mitochondrial organization and survival of activated Tregs. In addition, nonnuclear Notch1 activity is a positive regulator of Treg function, and perturbations of either Notch1 or autophagy can inhibit the suppressor activity of activated Tregs and result in immune hyperactivity. In contrast, another study specifically interrupting Notch signaling in Treg cells revealed that Notch signaling was a negative regulator for Treg polarization and function [40]. Researches on immune thrombocytopenia (ITP) and chronic hepatitis C have shown that there were no significant differences in Treg cell percentage, Foxp3 expression, and IL-10 production in DAPT-treated PBMCs [21, 23]. In this study, we isolated and cultured CD4^+^ T cells from PV patients; consistent with findings from ITP and hepatitis C patients, after DAPT treatment, no significant difference was found in expression levels of Treg cells, its specific transcription factor, and effective cytokine. Hence, the influence of Notch1 signaling on Treg cells and the response of Treg cells to Notch inactivation may depend on distinct experimental systems and diseases [41], and this needs to be clarified in the future.

## 6. Conclusions

In conclusion, the inhibitory function of Notch1 inactivation on Th17 cells is definite in PV and results in the reversal of an imbalanced ratio of Th17/Treg and their specific transcription factor ROR*γ*t/Foxp3, which provides novel insights into the potential therapeutic target of Notch signaling for PV.

## Figures and Tables

**Table 1:** List of primers for real-time quantitative RT-PCR.

Primers	Primer sequence (5′ to 3′)	Product length
Notch1	Forward: 5′-GTCAACGCCGTAGATGACC-3′	101 bp
	Reverse: 5′-TTGTTAGCCCCGTTCTTCAG-3′	

Hes-1	Forward: 5′-CTCTCTTCCCTCCGGACTCT-3′	186 bp
	Reverse: 5′-AGGCGCAATCCAATATGAAC-3′	

ROR*γ*t	Forward: 5′-CCTGGGCTCCTCGCCTGACC-3′	170 bp
	Reverse: 5′-TCTCTCTGCCCTCAGCCTTGCC-3′	

IL-17	Forward: 5′-TGTCCACCATGTGGCCTAAGAG-3′	119 bp
	Reverse: 5′-GTCCGAAATGAGGCTGTCTTTGA-3′	

Foxp3	Forward: 5′-CTGACCAAGGCTTCATCTGTG-3′	175 bp
	Reverse: 5′-ACTCTGGGAATGTGCTGTTTC-3′	

IL-10	Forward: 5′-GGACTTTAAGGGTTACCTGGGTTGCC-3′	105 bp
	Reverse: 5′-GCCTTGATGTCTGGGTCTTGGTTCTC-3′	

*β*-Actin	Forward: 5′-AGTTGCGTTACACCCTTTCTTG-3′	150 bp
	Reverse: 5′-TCACCTTCACCGTTCCAGTTT-3′	
